# Cross-validation Studies of a Novel Low-cost Hepatitis B Virus Quantitative Polymerase Chain Reaction System

**DOI:** 10.5005/jp-journals-10018-1255

**Published:** 2018-05-01

**Authors:** Jorge Aguiar, José A Silva, Gerardo García, Gerardo Guillén, Julio C Aguilar

**Affiliations:** 1 Department of Therapeutic Vaccine against Hepatitis B, Center for Genetic Engineering and Biotechnology (CIGB), Havana, Cuba; 2 Department of Oligonucleotide Synthesis, Center for Genetic Engineering and Biotechnology (CIGB), Havana, Cuba; 3 Department of Quality Control, Center for Genetic Engineering and Biotechnology (CIGB), Havana, Cuba

**Keywords:** Crossing point, Cycles threshold, Hepatitis B virus, Laboratory research, Plasmid, Primer, Quantitative polymerase chain reaction.

## Abstract

**Aim:**

This research focused on the results of the cross-validation program related with the performance of a Cuban novel low-cost real-time quantitative polymerase chain reaction (qPCR) assay for hepatitis B virus (HBV) quantification developed by the Therapeutic Vaccine against Hepatitis B Department, Vaccines Division, Center for Genetic Engineering and Biotechnology (CIGB), Havana, Cuba.

**Materials and methods:**

Dilution series with the plasmid standard at concentrations of 900,000 to 0.09 copies/reaction (c/r) were made for each PCR instrument. The mean cycles threshold (Ct) values and PCR efficiency were compared among the cyclers. Hepatitis B virus-positive serum samples were used for the calculation of reproducibility of the HBV assay. Biotecon Diagnostics (BCD) also ordered the oligo sequences from a second supplier and compared the PCR performance to those provided from the CIGB.

**Results:**

All PCR cyclers were able to detect concentrations up to 0.09 c/r. However, below the concentration of 9 c/r, the variation of results increased within and between the cyclers. The PCR efficiency showed satisfying results. The overall coefficient of variation (CV) cycler values were 1.29 and 0.91% for M6 and M19 respectively. No significance was observed between the different primer suppliers.

**Conclusion:**

The HBV assay was performed with a good concordance between the five real-time instruments from different suppliers. The HBV assay was also performed with a high reproducibility for samples with a high and a low viral load. The HBV assay is robust against different primer suppliers.

**How to cite this article:** Aguiar J, Silva JA, García G, Guillén G, Aguilar JC. Cross-validation Studies of a Novel Low-cost Hepatitis B Virus Quantitative Polymerase Chain Reaction System. Euroasian J Hepato-Gastroenterol 2018;8(1):38-41.

## INTRODUCTION

The HBV is the most common worldwide cause of viral hepatitis in humans. Over 400 million persons have become chronic carriers of the virus, and over 20% of them will develop hepatocellular carcinoma.^1^ Traditionally, the quantitative determination of the HBV level in the blood (viral load) is a basic variable in the follow-up and classification of the condition in patients with chronic hepatitis B. Currently, the viral load is the main variable used to measure the efficacy of therapeutic products for this disease.^2-4^ It is therefore, essential to develop a quantitative method to detect the amount of virus in the blood that would be equivalent to the current international systems validated for this purpose, minimizing the expenses for the health system and the patient.^3-7^

Years ago, we described the characteristics and validation experiments of a cheap and simple qPCR method^8^ that uses an unspecific and not very expensive commercial amplification kit (the Quantitect SYBR Green PCR kit, Qiagen, Germany). This was combined with the standards (based on a plasmid carrying the full-length HBV genome) and specific primers for the S gene, both produced at the CIGB, Havana, Cuba. The new combined kit was used to measure the concentration of HBV deoxyribonucleic acid (DNA) in serum samples of Cuban HBV carriers. The new low-cost qPCR combined system was compared with other commercial qPCR systems, such as “artus HBV LC PCR kit” (Qiagen) (R = 0.90) and “HBV Monitor system” (Roche) (R = 0.82).^8^ This demonstrated that the new system performed at the same level of quality than two of the most common international systems used for HBV DNA quantification.^8^

In addition, last year, we demonstrated that the oligonucleotide pair and the plasmid used for the standard curve in the new qPCR system for HBV DNA as reported by Aguiar et al^8^ are functionally very stable in severe working conditions (1-month incubation at 37°C).^9^ These results supported the robustness of the primer pair and the standard curve based on a plasmid carrying the complete HBV genome of 3.2 kb length from the aforementioned qPCR system for HBV viral load quantification.^8,9^

The present work summarizes the results of the cross-validation program related with the performance of a Cuban novel low-cost qPCR assay developed at the CIGB. The extent and design of this study were in agreement with a service provider with expertise in real-time PCR validation.

## MATERIALS AND METHODS

### Subjects

Serum HBV DNA from two Chronic hepatitis B (CHB) chronic patients (M6 and M19) was used for the calculation of reproducibility of the HBV assay, if using real samples with full-length viral genomes. Ten replicates were measured for each sample and instrument. One replicate had to be excluded for further calculations (Agilent Mx3005p-M6-n6).

### Hepatitis B Virus Deoxyribonucleic Acid Quantification

Hepatitis B Virus Deoxyribonucleic Acid Purification

The HBV DNA of the two CHB patients was purified from 200 μL of serum with the “QIAamp DNA Mini kit” (Qiagen, Germany), according to the manufacturer’s instructions.

Quantitative PCR Reaction

Quantitative PCR reaction was prepared as described previously by Aguiar et al^8^ using the unspecific commercial SYBR Green reaction mix from the “Quantitect SYBR Green PCR kit” (Qiagen, Germany).

Primers

The oligonucleotide pair used from two different suppliers at a concentration of 0.67 μM^8^ was previously published by other authors.^10,11^ The sequences of both are: sense 5’-GTGTCTGCGGCGTTTTATCA-3’ and anti-sense 5’-ACAAACGGGCAACATACCTT-3’. One supplier was the CIGB and the other supplier was BCD.

Thermal Cycling

Thermal cycling was performed in five real-time instruments from different suppliers: Applied Biosystems 7500 FAST, Roche LightCycler 480-II, Roche LightCycler 96, Agilent Mx3005p, Thermo Scientific PikoReal (24 well). Reaction conditions were 95°C for 15 minutes followed by 40 cycles of 94°C for 15 seconds, 58°C for 30 seconds, and 72°C for 30 seconds.

Standard Curve

The pST012012 plasmid (pST012012, plasmid bank of CIGB, 2012) that carries the complete 3.2 kb genome of the HBV was used as the quantification standard curve in the qPCR, as previously described by Aguiar et al^8^: It is employed as an eight-point standard curve: 9 × 10^5^ c/r, 9 × 10^4^ c/r, 9 × 10^3^ c/r, 9 × 10^2^ c/r, 90 c/r, 9 c/r, 0.9 c/r, and 0.09 c/r.

## RESULTS

### PCR Efficiency for Each PCR Instrument

Dilution series with the pST012012 plasmid were performed from 900,000 to 0.09 c/r for each of the five PCR instruments. Three replicates per dilution step were measured in the qPCR. The mean Ct values (Table 1) and PCR efficiency (Table 2) were compared between the cyclers.

**Table 1:** **Table 1:** Mean of Ct values for each PCR instrument

		*ABI 7500 FAST*		*LC 480-II*		*LC96*		*Mx3005p*		*PikoReal*		*Mean Ct*		*SD*		*CV (%)*	
900,000 c/r		15.40		15.55		16.90		15.81		14.49		15.63		0.87		5.54	
90,000 c/r		18.94		18.28		20.51		18.59		17.34		18.73		1.16		6.18	
9,000 c/r		22.71		21.93		24.27		22.77		20.84		22.50		1.26		5.59	
900 c/r		26.51		25.2		27.73		26.45		24.18		26.01		1.36		5.23	
90 c/r		29.55		27.81		31.25		30.03		26.95		29.12		1.73		5.94	
9 c/r		33.00		29.62		35.37		32.72		28.40		31.82		2.80		8.80	
0.9 c/r		34.82		29.96		36.86		35.38		29.18		33.24		3.44		10.36	
0.09 c/r		39.10		30.96		38.73		42.33		29.70		36.16		5.52		15.28	

SD: Standard deviation

**Table 2:** **Table 2:** Polymerase chain reaction efficiency for each PCR instrument

		*ABI 7500 FAST*		*LC 480-II*		*LC96*		*Mx3005p*		*PikoReal*	
Efficiency (%)		99.9		102.4		95.0		96.5		106.4	
R^2^		0.984		0.978		0.990		0.995		0.997	
Slope		–3.323		–3.265		–3.449		–3.409		–3.177	
Y-intercept		35.66		34.79		37.75		35.67		27.22	
Dynamic		Until		Until		Until		Until 0.9		Until	
range		0.09 c/r		9 c/r		0.9 c/r		c/r		90 c/r	

R^2^ Squared correlation or linear regression. Note that the dynamic range varies among the PCR instruments

### Inter- and Intrareproducibility

Hepatitis B virus-positive serum samples from chronic patients M6 and M19 were used for the calculation of reproducibility of the HBV assay, if using real samples with full-length viral genomes (Table 3). Cycles threshold values of 10 replicates were measured for each sample and instrument. One replicate had to be excluded for further calculations (Mx3005p-M6-n6).

### Second Supplier of Primer

Biotecon Diagnostics ordered the oligo sequences from a second supplier BCD and compared the qPCR performance with those provided from the CIGB. Dilution series with the pST012012 plasmid at concentrations of 900,000 to 0.09 c/r were used for the comparison on the Roche LightCycler^®^ 480 II. Three replicates per dilution step were measured in the qPCR. The mean of Ct or crossing point (Cp) values (Table 4) and PCR efficiency (Table 5) were compared.

## DISCUSSION

A previously reported validation program was generated, and certified by the Pedro Kouri Institute (IPK) in Havana, Cuba, in 2014.^8^ The present article summarizes the results of the cross-validation program conducted in collaboration with BCD GmbH, in order to verify the robustness of the in-house real-time PCR developed by the Therapeutic Vaccine against Hepatitis B Department at Vaccines Division, CIGB, Havana, Cuba.

**Table 3:** **Table 3:** Reproducibility of HBV assay

		*ABI 7500 FAST*				*LC 480-II*				*LC96*				*Mx3005p*				*PikoReal*			
*n*		*M6*		*M19*		*M6*		*M19*		*M6*		*M19*		*M6*		*M19*		*M6*		*M19*	
1		13.25		29.63		12.85		29.90		14.54		31.60		13.05		29.88		12.87		29.62	
2		13.22		29.44		12.74		30.24		14.49		31.81		12.79		30.02		12.77		29.68	
3		13.16		29.97		12.73		29.98		14.23		31.87		13.11		30.45		12.75		29.45	
4		13.08		30.05		12.74		29.67		14.29		31.77		12.96		30.11		13.00		29.40	
5		13.00		30.00		12.76		29.84		14.33		31.58		13.12		30.21		12.84		29.77	
6		12.99		30.03		12.85		29.51		14.79		31.56		42.12		29.47		12.82		30.11	
7		13.41		29.94		12.90		29.83		14.84		31.35		12.88		30.38		13.22		29.54	
8		13.30		30.36		12.72		29.75		15.09		32.38		13.14		30.20		12.98		29.48	
9		13.13		29.89		12.65		29.45		14.64		32.36		13.61		29.62		12.82		29.38	
10		13.25		30.28		12.64		29.52		14.75		31.75		13.13		30.19		12.75		29.67	
Mean Ct		13.18		29.96		12.76		29.77		14.60		31.80		13.09		30.05		12.88		29.61	
SD		0.13		0.27		0.09		0.24		0.27		0.33		0.23		0.31		0.15		0.22	
CV (%)		1.01		0.90		0.67		0.82		1.88		1.05		1.77		1.05		1.14		0.74	
M6 CV cycler (%)		1.29																			
M19 CV cycler (%)		0.91																			

Cycles threshold values from 10 replicates were evaluated for each sample and instrument; SD: Standard deviation; CV (%): Coefficient of variation expressed in %

**Table 4:** **Table 4:** Performance of the different primer suppliers (CIGB and BCD) on the Roche LightCycler® 480 II

		*Mean Cp*				*SD*				*CV (%)*			
*Concentration*		*CIGB*		*BCD*		*CIGB*		*BCD*		*CIGB*		*BCD*	
900,000 c/r		15.55		15.59		0.05		0.07		0.34		0.42	
90,000 c/r		18.28		18.43		0.05		0.14		0.28		0.75	
9,000 c/r		21.93		21.89		0.06		0.03		0.28		0.14	
900 c/r		25.2		25.14		0.12		0.14		0.46		0.55	
90 c/r		27.81		27.94		0.08		0.14		0.29		0.51	
9 c/r		29.62		29.51		0.12		0.27		0.39		0.92	
0.9 c/r		29.96		29.96		0.20		0.04		0.65		0.13	
0.09 c/r		30.96		30.55		0.49		0.04		1.58		0.14	

Dilution series with the pST012012 plasmid at concentrations of 900,000 to 0.09 c/r were used, and three replicates per dilution step were measured in the qPCR. The means of Ct or Cp values were compared between the thermocyclers. SD: Standard deviation; CV (%): Coefficient of variation expressed in percent

**Table 5:** **Table 5:** Performance of the different primer suppliers (CIGB and BCD) on the Roche LightCycler® 480 II

		*CIGB*		*BCD*	
Efficiency (%)		102.4		108.1	
R^2^		0.978		0.986	
Slope		–3.265		–3.142	
Y-intercept		34.79		34.23	
Dynamic range		Until 9 c/r		Until 9 c/r	

PCR: Polymerase chain reaction efficiency expressed in percent was compared between the primer suppliers; R^2^: Squared correlation

The in-house qPCR HBV assay developed by CIGB performed in good concordance with the five real-time instruments studied from different suppliers. All cyclers were able to detect up to 0.09 c/r. However, below the concentration of 9 c/r the variation of results increased within and between the cyclers. Thus, the dynamic range had to be adapted for each cycler manually (Table 1). The PCR efficiency (expressed in %) between the five thermocyclers was calculated based on the dynamic range and showed satisfying results (Table 2).

Also, the high values of the squared correlation or linear regression (R^2^) observed in Table 2 can be used for a precise calculation of HBV loads in specimens within the dynamic range, because R^2^ is a statistic that will give some information about the goodness of fit of a model.^12^ In regression, the R^2^ coefficient of determination is a statistical measure of how well the regression line approximates the real data points. An R^2^ of 1 indicates that the regression line perfectly fits the data.^12^

In addition, serum HBV from two CHB chronic patients (M6 and M19) was used for the calculation of the reproducibility of the CIGB-HBV assay for the verification of the performance in the cases that use real samples with full-length viral genomes (Table 3). The overall CV cycler values were 1.29 and 0.91% for M6 and M19 respectively (Table 3), which means that the CIGB-HBV assay performed with a high reproducibility for samples with a high (M6) and a low (M19) viral load.

Finally, BCD also ordered the oligo sequences from a second supplier and compared the CIGB qPCR performance with those primers provided from the CIGB (Tables 4 and 5). No differences in the Cp values were observed between the primer suppliers. Thus, the qPCR HBV assay for the detection of HBV in serum developed by the CIGB is robust against different oligonucleotide suppliers.
